# Nutritional Profile and Chemical Stability of Pasta Fortified with Tilapia (*Oreochromis niloticus*) Flour

**DOI:** 10.1371/journal.pone.0168270

**Published:** 2016-12-14

**Authors:** Maria Lúcia G. Monteiro, Eliane T. Mársico, Manoel S. Soares, Amanda O. Magalhães, Anna Carolina V. C. S. Canto, Bruno R. C. Costa-Lima, Thiago S. Alvares, Carlos A. Conte

**Affiliations:** 1 Departament of Food Technology, Universidade Federal Fluminense, Rio de Janeiro, Brazil; 2 Departament of Food Engineering, School of Agronomy, Universidade Federal de Goiás, Goiânia, Brazil; 3 Institute of Nutrition, Universidade Federal do Rio de Janeiro, Rio de Janeiro, Brazil; 4 Institute of Chemical, Universidade Federal do Rio de Janeiro, Rio de Janeiro, Brazil; Istituto di Biologia e Biotecnologia Agraria Consiglio Nazionale delle Ricerche, ITALY

## Abstract

Physicochemical parameters of pasta enriched with tilapia (*Oreochromis niloticus*) flour were investigated. Five formulations were prepared with different concentrations of tilapia flour as partial substitute of wheat flour: pasta without tilapia flour (PTF0%), pasta with 6% (PTF6%), 12% (PTF12%), 17% (PTF17%), and 23% (PTF23%) of tilapia flour. The formulations were assessed for proximate composition, fatty acid and amino acid profile on day 1 whereas, instrumental color parameters (L*, a* and b* values), pH, water activity (a_w_), and lipid and protein oxidation were evaluated on days 1, 7, 14, and 21 of storage at 25°C. Fortification with tilapia flour increased (*p* < 0.05) protein, lipid, ash, total essential amino acids, and total polyunsaturated fatty acids contents. In addition, supplementation of pasta with tilapia flour decreased (*p* < 0.05) lightness and water activity while redness, yellowness, pH values, and lipid oxidation were increased (*p* < 0.05) in a level-dependent manner. Nevertheless, all formulations were exhibited storage stability at 25°C. In general, protein oxidation was greater (*p* < 0.05) in the pasta containing 12%, 17%, and 23% of tilapia flour than their counterparts, and the storage promoted an increase (*p* < 0.05) on the carbonyl content in all formulations. Thus, pasta with 6% of tilapia flour has the potential to be a technological alternative to food industry for the nutritional enrichment of traditional pasta with negligible negative effects on the chemical stability of the final product during 21 days at 25°C.

## Introduction

The global pasta consumption is expanding in recent years with reports documenting an increase of 1 million ton in 2013 in relation to 2012 [1,2], as a consequence of nutritional aspects, favorable taste, as well as due to the convenient storage, cooking and preparation [3]. Although pasta contains high carbohydrate level (75% of weight; [4]), it is considered a low glycemic index product (≤ 55; [5]) and a potential food target for nutritional fortification [6]. Furthermore, food industry is constantly developing strategies to reduce the manufacture costs, increase profitability, and improve the nutritional quality of products. In this matter, the incorporation of low glycemic index ingredients into wheat based food products represents a promising strategy to address the demand for healthy products and also attend a particular niche comprised by consumers with a specific disease such as diabetes mellitus and cardio-vascular diseases [7].

Fish flour is a by-product from fish processing and represent a cheap source of high-quality nutrients for the human diet, mainly due to high levels of essential amino acids and polyunsaturated fatty acids [8,9]. Furthermore, in contrast with pasta, the fish matrix has very low content of carbohydrates [10] thus, very low glycemic index. Therefore, fish flour is potentially an interesting subject for wheat flour replacement in bakery products [11]. Among freshwater fish species produced worldwide, Nile tilapia (*Oreochromis niloticus*) contributes with approximately 9% of the total amount of fish produced [12] however, this specie exhibits low dressing yield (roughly 30% of the live weight), and its fillets are highly perishable when marketed as fresh fillets [11,13]. On the other hand, tilapia flour is manufactured from about 29% of the live weight representing a potential by-product from tilapia processing, and demonstrates a reasonable stability during storage at 25°C [9].

Previous studies observed the nutritional and physicochemical aspects of dried pasta manufactured with the mince and the oil of *Sardinella longiceps* [14], *Catla Catla* mince [15], and fresh pasta enriched with protein concentrate of tilapia [16]. However, as nutritional composition and chemical stability vary depending on fish species and the processing procedure [4,17,18], the physicochemical properties of dried pasta manufactured with partial replacement of wheat flour by tilapia flour is still unknown. In this context, the objectives of the present study were (1) to investigate the nutritional value of pasta manufactured with partial replacement of wheat flour by tilapia flour as partial replacement of wheat flour, and (2) to evaluate their chemical stability during 21 days at 25°C.

## Materials and Methods

### Tilapia Flour Preparation

A total of 9.0 ± 0.2 kg of mechanically separated meat (MSM) of tilapia was purchased from a commercial fish farm (Cooperativa Regional de Piscicultores e Ranicultores do Vale do Macacu e Adjacências Ltda.) located in Rio de Janeiro, Brazil (22°33'58.3"S 42°41'48.2"W). The MSM was packed in low density polyethylene bags, frozen at –80°C, and transported during 3 h in a styrofoam box containing ice to the bakery pilot plant of the Department of Food Engineering, Universidade Federal de Goiás, Goiânia, Brazil. The tilapia flour was obtained by drying the MSM for 12 hours at 65°C in a forced-air convection oven (TE-394/3, Tecnal, Piracicaba, SP, Brazil), and tilapia flour was obtained.

### Pasta Preparation

Five pasta formulations (PF) were prepared using different wheat flour (WF) and tilapia flour (TF) levels (w/w) as following: 58 g of WF, 52 g of WF plus 6 g of TF, 46 g of WF plus 12 g of TF, 41 g of WT plus 17 g of TF, and 35 g of WF plus 23 g of TF, representing 0%, 10.34%, 20.68%, 29.31%, and 39.65% of WF replacement by TF, respectively. All formulations were optimized to 37% of added water and 5% of whole powdered egg before the drying step (Table 1) and referred to as PTF0% (pasta without tilapia flour), PTF6% (pasta with 6% of tilapia flour), PTF12% (pasta with 12% of tilapia flour), PTF17% (pasta with 17% of tilapia flour), and PTF23% (pasta with 23% of tilapia flour). White wheat flour (*Triticum aestivum* L.) and whole powdered egg were purchased from a local market in Goiânia, Brazil. All ingredients were manually mixed and the dough formed was drawn between rolls of a pasta machine with a gap of 4 mm (Anodilar, Caxias do Sul, RS, Brazil), and cut into 260 mm length and 5 mm width. Then, fresh pasta was dried in a forced-air convection oven (TE-394/3, Tecnal, Piracicaba, SP, Brazil) at 45°C for 2 hours.

**Table 1:** Pasta formulations with different tilapia flour levels.

Ingredients (%)	Formulations				
	PTF0%	PTF6%	PTF12%	PTF17%	PTF23%
Wheat flour	58	52	46	41	35
Tilapia flour	0	6	12	17	23
Whole powdered egg	5	5	5	5	5
Water	37	37	37	37	37

PTF0%, PTF6%, PTF12%, PTF17%, and PTF23% mean pasta with 0%, 6%, 12%, 17%, and 23% (w/w) of tilapia flour, respectively.

### Samples Preparation

All formulations were aerobically packaged and transported (< 3 h) to the Department of Food Technology, Universidade Federal Fluminense, Rio de Janeiro, Brazil. Samples were analyzed for proximate composition, fatty acids profile, and amino acid content on day 1 whereas, instrumental color parameters (L*, a*, and b* values), water activity (a_w_), pH, and lipid and protein oxidation were analyzed on days 1, 7, 14 and 21 of storage at 25°C. Prior to all analysis, samples were homogenized in a commercial blender (Oster Co., Milwaukee, WI, USA) in order to standardization of the samples. The whole experiment, consisting of tilapia MSM and pasta manufacture followed by physicochemical analyses, was repeated three times (n = 3).

### Proximate Composition

The moisture, protein, and ash contents were determined at day 1 according to methods described by the Association of Official Analytical Chemists [19]. For the determination of moisture, samples were dried at 100−102°C until constant weight (AOAC method 950.46B). The protein content was estimated using Kjeldahl procedure (conversion factors equal to 6.25 for pasta manufactured with tilapia flour, and 5.70 for pasta formulated without tilapia flour [20]; AOAC method 955.04), and the ash content was evaluated by sample incineration in a muffle furnace at 550°C (AOAC method 920.153 [19]). The total lipid content was cold-extracted using a modified methanol:chloroform solvent (2:1 v/v) optimized to 80% of moisture [21]. The carbohydrate content was estimated as the difference between 100% and the sum of the contents of moisture, protein, ash, and lipids; and the energy value was calculated using the formula described by Merrill and Watt [22]:

Energy value (kcal/100g) = 4 × protein (%) + 9 × lipid (%) + 4 × carbohydrate (%)

All analyses were performed in triplicate.

### Fatty Acid Profile

The fatty acids present in the cold-extracted methanol:chloroform [21] layer were acid methylated [23–25], and analyzed in a gas chromatograph coupled with a flame ionization detector (Perkin Elmer, Waltham, MA, USA). The fatty acid methyl esters were separated in an Omegawax-320 column (30 m long, 0.32 mm internal diameter and 0.25 mm film thickness; Sulpeco, USA). The injected sample volume was 2 μL and the temperature of the injector (split of 1:20) and the detector temperatures were 260°C and 280°C, respectively. The column temperature started at 110°C with a ramp of 40°C/min until 233°C which was held for 2 min. After that, the oven temperature reached 240°C at 1°C/min and held for 21 min. The identification of the different fatty acid methyl esters was based on the specific retention times obtained from a mixture containing 37 different standards (Supelco 18919-1AMP, Sigma-Aldrich, St. Louis, MO, USA). Helium was utilized as the carrier gas with a flow rate of 1.8 mL/min at 10 psi.

### Amino Acid Profile

The protein hydrolysis was performed at day 1 according to protocol described by Chlou and Wang [26] and Marconi et al. [27] with some modifications while the derivatization and chromatographic conditions were conducted as described by Gatti et al. [28] with slight changes. Aliquots of 100 mg of each sample was homogenized with 5 mL of 6 M hydrochloric acid in digestion vessels flushed with nitrogen for 1 min to remove oxygen. These vessels were subjected to 800 W of microwave power in a DGT Plus microwave oven (Provecto Analítica, Sao Paulo, Brazil) for 1 min, and then 5 mL of 75.95 mM sodium citrate solution (pH 2.2) was added followed by centrifugation at 24,000 × *g* for 10 min. The supernatant was filtered through Whatman paper N°1, and fifty microliters were derivatized with 40 μL of 45 mM 2,5-dimethyl-1H-pyrrole-3,4-dicarbaldehyde (DPD) for 10 min at 25°C. After derivatization, an aliquot of 300 μL of water was added, and then 20 μL was injected into the high performance liquid chromatograph (Shimadzu, Tokyo) equipped with a Phenomenex Gemini 5 μm ODS (250 mm × 3.0 mm i.d.) column. Amino acids were separated at 33°C with an A:B gradient wherein A was methanol and B was 50 mM triethylammonium phosphate buffer (pH 2.5) at a flow-rate of 0.32 mL/min. The gradient profile was set as follows: t_0min_ 8% A, t_10min_ 32% A, t_25min_ 50% A, and t_30min_ 8% A. The amino acids were detected utilizing a diode array detector at 320 nm, and quantified through calibration curves of each amino acid analyzed. The amino acids analysis was performed in duplicate.

### Instrumental Color Parameters

The instrumental color parameters were determined using a Minolta CM-600D Spectrophotometer (Minolta Camera Co., Osaka, Japan). An aliquot of 25 g was placed into a polystyrene petri dishes (60 mm × 15 mm), and subsequently, CIE L* (lightness) a* (redness) b* (yellowness) values were recorded from four distinct sampling areas from the air-exposed surface utilizing illuminant A, 8 mm aperture, and 10° observer at 25°C. Prior to color measurements, the spectrophotometer was calibrated following standard protocol described in American Meat Science Association [29]. L*, a* and b* values were determined in quadruplicate.

### Water Activity (a_w_) and pH

Five grams of sample were transferred to a disposable sample cup (Decagon Devices, Pullman, WA, USA) and the a_w_ value was directly recorded at 25°C in a Pawkit water activity meter (Decagon Devices, Pullman, WA, USA) according to manufacturer guidelines. The pH values were determined after homogenizing 10 g of sample in 90 mL of distilled water using a penetration electrode of a digital pH meter (Hanna Instruments, Woonsocket, USA) [30]. Prior to analysis, the pH meter apparatus was calibrated using buffered solutions (pH = 4.00 and pH = 7.00). Pasta a_w_ and pH were determined in triplicate and quadruplicate, respectively on each storage day analyzed.

### Lipid and Protein Oxidations

Lipid oxidation was evaluated by the thiobarbituric acid-reactive substances (TBARS) method proposed by Yin et al. [31], whereas protein oxidation was estimated based on the estimation of carbonyl contents as described by Oliver et al. [32] with some modifications [33,34]. Both TBARS and carbonyl analyzes were performed in duplicate on each day of storage.

For the TBARS measurement, an aliquot of 5 g of sample was homogenized with 22.5 mL of 11% trichloroacetic acid for 1 min in a T18 basic Ultra-Turrax at 11,000 rpm (IKA, Wilmington, USA) followed by 1 min in an ice bath, and another 1 min of homogenization at equal conditions. The homogenate was filtered using Whatman paper N°1, and 1 mL of 20 mM TBA was added to 1 mL of the filtrate following incubation in dark conditions for 20 h at 25°C. The absorbance value at 532 nm was read using a UV-1800 spectrophotometer (Shimadzu, Kyoto, Japan) and the results were expressed as TBARS number.

For determination of carbonyl content, 3 g of sample was homogenized with 0.15 M KCl (pH 7.4) using a T18 basic Ultra-Turrax at 7,100 rpm (IKA, Wilmington, USA) for 90 s. The proteins present in the homogenate were precipitated with 10% TCA, and centrifuged (ST 16R, Thermo Scientific, Wilmington, DE) at 5,000 × *g* for 5 min at 4°C. After discarding the supernatant 10 mM 2,4-dinitrophenylhydrazine (DNPH) in 2 N HCl was added to the precipitate and incubated for 1 h at 25°C in the dark with brief vortexing every 15 min. Then the DNPH-reacted substrate was precipitated with 10% TCA following centrifugation at 11,000 × *g* for 10 min at 4°C. The precipitate was washed three times with 1:1 (v/v) ethanol/ethyl acetate solution and then solubilized with 6M guanidine hydrochloride in 20 mM sodium phosphate buffer (pH 6.5). A final centrifugation at 11,000 × *g* for 10 min at 25°C was employed to remove insoluble particles. The absorbance value was measured at 370 nm using a UV-1800 spectrophotometer (Shimadzu, Kyoto, Japan), and carbonyls concentration was calculated using an absorptivity coefficient for the protein hydrazones of 21.0 × mM^-1^ × cm^-1^ for the protein hydrazones. The results were expressed as nmol of carbonyl/mg of protein. For protein content measurement, the aforementioned steps were performed with 2N HCl instead of the DNPH solution, and the absorbance value at 280 nm was recorded from the homogenate solubilized in 6M guanidine hydrochloride. The results were expressed as nmol of carbonyl/mg of protein.

### Statistical Analysis

The influence of partial replacement of wheat flour by tilapia flour (PTF0%, PTF6%, PTF12%, PTF17%, and PTF23%) and storage period (1, 7, 14, and 21 days) at 25°C were separately analyzed using one-way ANOVA followed by Tukey test (*p* < 0.05). Data of proximate composition were also analyzed by linear regression. These analyses were carried out utilizing XLSTAT software, version 2012.6.08 (Addinsoft, New York, NY, USA). In addition, the data of instrumental color parameters (L*, a*, and b* values), pH, a_w_, lipid and protein oxidations were plotted using SigmaPlot software, version 11 (Systat Software Inc., San Jose, CA). The whole experiment comprising the manufacture of tilapia flour and pasta formulations, and physicochemical analyses were repeated three times (n = 3).

## Results and Discussion

### Proximate Composition

The addition of tilapia flour demonstrated a level-dependent effect (*p* < 0.05) on the proximate composition of the pasta formulations (Table 2). The tilapia flour addition decreased (*p* < 0.05) moisture and carbohydrate contents whereas increased (*p* < 0.05) the levels of lipid, protein, and ash, potentially due to the tilapia flour compositions. While tilapia flour exhibits low carbohydrate level (< 1.5%) and increased protein (> 45%), lipid (> 25%) and ash (> 3%) contents [11,35], wheat counterpart contains increased amount of carbohydrate (> 75%) and is low in protein (< 11%), lipid (≤ 1.5%) and ash (≤ 0.38%) [4,36]. These observations explain the greatest lipid, protein, and ash contents, as well as lower carbohydrate levels in pasta formulations enriched with tilapia flour (PTF23% > PTF17% > PTF12% > PTF6%) when compared to control counterparts. Moreover, the decrease in the moisture content promoted by the tilapia flour addition can be attributed to a greater protein-polysaccharides interaction when compared to wheat counterparts, which under heating conditions results in protein denaturation favoring intermolecular network [37,38]. The interaction between polysaccharides and proteins through electrostatic forces promotes the entrapment of water, and subsequently a more homogeneous network with less free water [38,39] which is associated with a decrease on the moisture content in foods rich in proteins and polysaccharides [40].

**Table 2:** Proximate composition (%) and energy value (kcal/100g) in pasta fortified with different tilapia flour levels during 21 days at 25°C.

Formulations	Parameters					
	Moisture	Lipid	Protein	Ash	Carbohydrate	Energy value
PTF0%	7.69±0.28^a^	3.21±0.17^e^	15.77±0.84^e^	0.69±0.03^e^	72.28±1.24^a^	380.99±3.46^c^
PTF6%	7.18±0.12^b^	3.64±0.17^d^	25.16±1.22^d^	1.13±0.07^d^	62.45±1.99^b^	383.33±2.70^c^
PTF12%	6.25±0.27^c^	4.18±0.21^c^	32.06±1.19^c^	1.44±0.02^c^	56.02±1.11^c^	389.94±1.83^b^
PTF17%	5.64±0.34^d^	4.65±0.25^b^	41.00±0.29^b^	1.77±0.05^b^	46.69±0.66^d^	392.60±3.93^b^
PTF23%	4.42±0.22^e^	5.45±0.28^a^	47.91±0.11^a^	2.06±0.06^a^	40.83±0.94^e^	404.04±5.91^a^

PTF0%, PTF6%, PTF12%, PTF17%, and PTF23% mean pasta with 0%, 6%, 12%, 17%, and 23% (w/w) of tilapia flour, respectively. Results are expressed as means ± standard deviation (n = 3). Different letters indicate differences (*p* < 0.05) among formulations.

In agreement with our results, Devi et al. [15], Anbudhasan et al. [14], and Goes et al. [16] reported an increase on protein, ash, and lipid contents when *Catla Catla* mince, *Sardinella longiceps* mince and oil, and tilapia protein concentrate were added to pasta formulations, respectively. Furthermore, Devi et al. [15] and Goes et al. [16] reported lower carbohydrate level in pasta manufactured with *Catla catla* mince and tilapia protein concentrate, respectively, when compared to control counterparts. On the other hand, although Devi et al. [15] and Anbudhasan et al. [14] reported greater moisture values in fish-enriched pasta, Goes et al. [16] did not find difference in moisture content between pasta formulations with and without fish protein concentrate. The variation in moisture values of pasta enriched with fish by-products in literature can be attributed mainly to different formulations and processing procedures [14].

Pasta formulation with 23% of tilapia flour (PTF23%) exhibited the greatest (*p* < 0.05) energy value. No difference (*p* > 0.05) was observed between PTF17% and PTF12%, as well as between PTF6% and PTF0%. In addition, PTF17% and PTF12% presented greater (*p* < 0.05) energy value than PTF6% and PTF0%. Our findings of energy value can be explained by the changes on the lipid, protein, and carbohydrate contents promoted by tilapia flour addition in association to their respective individual weights in the formula proposed by Merrill and Watt [22]. Although pasta formulations containing 12%, 17% and 23% of tilapia flour replacement presented greater (*p* < 0.05) energy value than control, fish by-products exhibit high quality of nutrients such as polyunsaturated fatty acids and essential amino acids [8,9] which are limited in wheat-based foodstuff [4,41]. Based on our findings (Table 3), tilapia flour addition increased (*p* < 0.05) the contents of protein, lipid, ash contents and the energy value, whereas it decreased (*p* < 0.05) moisture and carbohydrate level. Furthermore, carbohydrate and protein were the compounds affected in greatest extent (high slope value) by the tilapia flour inclusion.

**Table 3:** Linear regression coefficients of proximate composition (%) and energy value (kcal/100g) of the pasta formulations as influenced by the ratio of tilapia:wheat flours.

Parameter	Coefficients			
	y-intercept	slope	p-value	r-squared
Moisture	7.66±0.08	-4.97±0.20	<0.0001	0.9580
Lipid	3.26±0.06	3.37±0.17	<0.0001	0.9350
Protein	18.80±0.72	47.50±1.92	<0.0001	0.9580
Ash	0.82±0.04	2.09±0.11	<0.0001	0.9399
Carbohydrate	68.66±0.91	-45.82±2.34	<0.0001	0.9388
Energy value	380.33±0.61	33.71±1.85	<0.0001	0.8685

### Fatty Acid Profile

A total of fifteen fatty acids were identified (Table 4) in tilapia flour-enriched pasta. Regarding saturated fatty acids (SFA), pasta manufactured with tilapia flour exhibited greater (*p* < 0.05) amount of pentadecylic (C15:0), palmitic (C16:0), stearic (C18:0) and heneicosylic acids (C21:0) whereas, lower (*p* < 0.05) myristic acid (C14:0) level than pasta made without tilapia flour. Nonetheless, no difference (*p* > 0.05) was observed in total SFA amongst all formulations. With regards to monounsatured fatty acids (MUFA) tilapia flour fortification increased (*p* < 0.05) the contents of palmitoleic (C16:1) and paullinic acids (C20:1), and decreased (*p* < 0.05) the amount of the heptadecenoic (C17:1) and vaccenic acids (C18:1) resulting on a decrease (*p* < 0.05) of total MUFA content in tilapia flour-added pastas when compared to control. Nonetheless, all the identified polyunsaturated fatty acids (PUFA) exhibited an increased (*p* < 0.05) due to tilapia flour addition leading to the greatest (*p* < 0.05) total PUFA level in PTF23% and PTF17% followed by PTF12%, PTF6%, and the lowest (*p* < 0.05) value reported in the PTF0% formulation.

**Table 4:** Fatty acid profile (g of individual fatty acids/100g of total fatty acid) of pasta fortified with different tilapia flour levels.

Fatty acids	PTF0%	PTF6%	PTF12%	PTF17%	PTF23%
C14:0	43.87±2.46^a^	29.74±1.35^b^	23.22±1.10^c^	22.21±1.16^c^	21.92±1.75^c^
C15:0	0.00±0.00^d^	2.06±0.13^c^	4.81±0.00^b^	4.41±0.33^b^	7.35±0.28^a^
C16:0	16.29±0.47^b^	19.22±0.92^a^	20.39±0.64^a^	21.09±2.08^a^	19.77±0.48^a^
C16:1	0.00±0.00^c^	0.00±0.00^c^	3.64±0.15^b^	3.90±0.38^b^	4.74±0.45^a^
C17:1	2.99±0.25^a^	2.13±0.02^b^	1.76±0.09^b^	1.10±0.09^c^	1.12±0.11^c^
C18:0	10.65±0.94^c^	20.63±1.90^ab^	24.22±0.35^a^	23.12±0.68^ab^	19.58±1.55^b^
C18:1	22.40±0.27^a^	16.41±0.11^b^	13.11±1.23^c^	11.79±0.36^c^	11.25±0.10^c^
C18:2	0.00±0.00^d^	1.59±0.10^c^	2.52±0.22^b^	3.32±0.30^a^	3.51±0.26^a^
C18:3n3	0.00±0.00^c^	0.00±0.00^c^	0.28±0.00^b^	0.34±0.04^a^	0.28±0.00^b^
C18:3n6	0.00±0.00^c^	0.57±0.05^b^	0.66±0.00^b^	1.91±0.14^a^	1.86±0.07^a^
C20:1	0.00±0.00^c^	0.00±0.00^c^	0.97±0.00^b^	1.21±0.02^a^	1.13±0.11^ab^
C20:3n3	0.00±0.00^c^	1.27±0.12^b^	1.54±0.13^ab^	1.65±0.16^a^	1.54±0.11^ab^
C21:0	1.17±0.13^d^	1.81±0.13^c^	2.47±0.25^b^	2.89±0.07^b^	5.97±0.06^a^
C22:1n9	0.00±0.00^c^	0.00±0.00^c^	0.26±0.00^ab^	0.25±0.02^b^	0.29±0.02^a^
C22:2	0.00±0.00^b^	0.69±0.07^a^	0.65±0.06^a^	0.60±0.06^a^	0.66±0.06^a^
∑ SFA	71.99±1.78^a^	73.46±1.67^a^	75.12±0.12^a^	73.72±2.08^a^	74.59±0.55^a^
∑ MUFA	25.39±0.10^a^	18.54±0.12^b^	19.48±1.31^b^	18.21±0.03^b^	18.29±0.52^b^
∑ PUFA	0.00±0.00^d^	4.12±0.04^c^	5.91±0.05^b^	8.07±0.19^a^	8.14±0.56^a^

PTF0%, PTF6%, PTF12%, PTF17%, and PTF23% mean pasta with 0%, 6%, 12%, 17%, and 23% (w/w) of tilapia flour, respectively. ∑ SFA: sum of saturated fatty acid; ∑ MUFA: sum of monounsaturated fatty acid; ∑ PUFA: sum of polyunsaturated fatty acid. Results are expressed as means ± standard deviation (n = 3). Different letters indicate differences (*p* < 0.05) among formulations.

Studies evaluating the fatty acid profile of pasta products fortified with fish by-products are limited and there are no reports regarding fatty acid composition in dried pasta enriched with tilapia meat and/or tilapia flour at the present moment. Our findings can be attributed to the difference between fatty acid composition of wheat flour and tilapia flour [14,42]. According to Anbudhasan et al. [14], pasta products contain mostly carbohydrates and exhibit low fatty acid content. On the other hand, tilapia flour presents elevated levels of MUFA and PUFA [42]. The lack of difference (*p* > 0.05) in total SFA level, and the decrease (*p* < 0.05) in MUFA contents followed by tilapia flour addition is potentially due to the binding of amylose to fatty acids [43,44] hindering their extraction by methanol:chloroform. Zhou et al. [44] observed that fatty acids are introduced into starch granule and SFA are more prone to be involved with the formation of lipid-amylose complex due to absence of double bonds. Furthermore, these authors suggested that the increase on unsaturation within the fatty acid chain negatively influences lipid-amylose complexation which potentially explains the results of the present study. In partial agreement with our findings, Anbudhasan et al. [14] reported an increase on the contents of myristic, palmitic, palmitoleic, stearic, linolenic, behenic, eicosapentaenoic, and docosahexaenoic acids, as well as a decrease in oleic, linoleic, and arachidic acids levels when sardine meat was added to pasta formulations.

### Amino Acids Composition

Sixteen amino acids were identified (Table 5) in the present study. Tilapia flour addition increased (*p* < 0.05) the levels of essential amino acids (EAA) such as histidine, lysine, threonine, methionine, valine, and leucine, and some non-essential amino acids (NEAA) including arginine, serine, glutamine, glycine, aspartic acid, glutamic acid, alanine, and tyrosine. Amongst the EAA, histidine and lysine levels were increased (*p* < 0.05) in a level-dependent manner of tilapia flour inclusion. While, the contents of threonine and valine were higher (*p* < 0.05) in the formulations containing 12%, 17% and 23% of tilapia flour, methionine and leucine levels were increased (*p* < 0.05) by tilapia flour inclusions greater or equal to 6% and 17%, respectively. No difference (*p* > 0.05) was observed in the tryptophan and phenylalanine contents. Amongst the NEAA, arginine, serine, glutamine, glycine, and tyrosine levels were increased (*p* < 0.05) by tilapia flour fortifications greater than 6%; whereas, glutamic acid content was greater (*p* < 0.05) in PTF17% than in PTF6% and PTF0%. Similarly, aspartic acid and alanine contents exhibited an increase (*p* < 0.05) due to the addition of tilapia flour. Pasta manufactured with tilapia flour demonstrated greater (*p* < 0.05) total EAA and NEAA contents than the control counterpart and the increase exhibited a tilapia flour inclusion level-dependent manner (PTF23% > PTF17% > PTF12% > PTF6% > PTF0%).

**Table 5:** Amino acid profile (mg of individual amino acids/g of tilapia pasta) of pasta fortified with different tilapia flour levels.

Amino acids	PTF0%	PTF6%	PTF12%	PTF17%	PTF23%
**Essentials**					
Histidine	0.16±0.01^d^	0.23±0.01^c^	0.47±0.01^b^	0.63±0.00^a^	0.93±0.03^a^
Lysine	0.18±0.01^d^	0.31±0.02^c^	0.49±0.04^b^	0.79±0.07^a^	1.23±0.02^a^
Threonine	0.13±0.01^c^	0.19±0.00^c^	0.28±0.03^b^	0.52±0.05^a^	0.62±0.01^a^
Methionine	0.11±0.01^d^	0.15±0.00^c^	0.18±0.01^bc^	0.21±0.02^ab^	0.25±0.01^a^
Valine	0.21±0.01^b^	0.28±0.02^b^	0.38±0.03^a^	0.37±0.03^a^	0.40±0.03^a^
Tryptophan	0.11±0.00^a^	0.11±0.00^a^	0.11±0.00^a^	0.12±0.01^a^	0.12±0.01^a^
Phenylalanine	0.20±0.01^a^	0.20±0.01^a^	0.20±0.01^a^	0.22±0.01^a^	0.22±0.01^a^
Leucine	0.35±0.02^b^	0.35±0.02^b^	0.35±0.02^b^	0.50±0.02^a^	0.50±0.02^a^
**Non-essentials**					
Arginine	0.41±0.03^c^	0.53±0.02^bc^	0.66±0.06^b^	0.90±0.01^a^	1.06±0.08^a^
Serine	0.24±0.02^c^	0.26±0.00^c^	0.41±0.03^b^	0.70±0.01^a^	0.69±0.07^a^
Glutamine	0.08±0.01^d^	0.10±0.00^cd^	0.11±0.01^bc^	0.13±0.01^ab^	0.14±0.01^a^
Glycine	0.12±0.00^c^	0.89±0.01^c^	2.27±0.08^b^	5.06±0.03^a^	6.24±0.54^a^
Aspartic acid	0.57±0.06^b^	0.86±0.01^a^	1.05±0.04^a^	0.99±0.04^a^	2.35±0.19^a^
Glutamic acid	0.98±0.07^c^	1.21±0.09^c^	1.50±0.11^bc^	2.30±0.19^ab^	2.73±0.10^a^
Alanine	0.93±0.10^c^	1.32±0.12^b^	1.57±0.07^b^	2.12±0.08^a^	2.42±0.02^a^
Tyrosine	0.14±0.02^c^	0.18±0.00^bc^	0.21±0.00^b^	0.30±0.03^a^	0.35±0.02^a^
∑ EAA	1.84±0.01^d^	2.21±0.00^c^	2.85±0.05^b^	3.54±0.10^a^	4.45±0.02^a^
∑ NEAA	3.47±0.16^d^	5.35±0.05^c^	7.78±0.03^b^	12.50±0.00^a^	15.98±0.75^a^

PTF0%, PTF6%, PTF12%, PTF17%, and PTF23% mean pasta with 0%, 6%, 12%, 17%, and 23% (w/w) of tilapia flour, respectively. ∑ EAA: sum of essential amino acids; ∑ NEAA: sum of non-essential amino acids. Results are expressed as means ± standard deviation (n = 3). Different letters indicate differences (*p* < 0.05) among formulations.

The amino acid profile of pasta products enriched with protein sources is scarce in the literature and the amino acid composition of dried pasta fortified with tilapia flour is still unknown. Pasta products manufactured with wheat contain a limited amount of essential amino acids representing a potential target for the incorporation of protein sources such as fish by-products [15] which are rich in essential amino acids [8]. In agreement with our results, Shogren et al. [45] and Ogur [46] reported similar pattern for total EEA and NEAA levels in soybean-fortified spaghetti and bread enriched with *Tinca tinca* mince, respectively. On the other hand, these authors also reported an increase on tryptophan and phenylalanine contents due to soybean and *T*. *tinca* fortification, which disagrees with our present findings. According to Gaie-Levrel et al. [47], tryptophan and phenylalanine are considered thermolabile α-amino acids which potentially explains the observed levels of these amino acids in the present study.

### Instrumental Color Parameters

**Fig 1:**
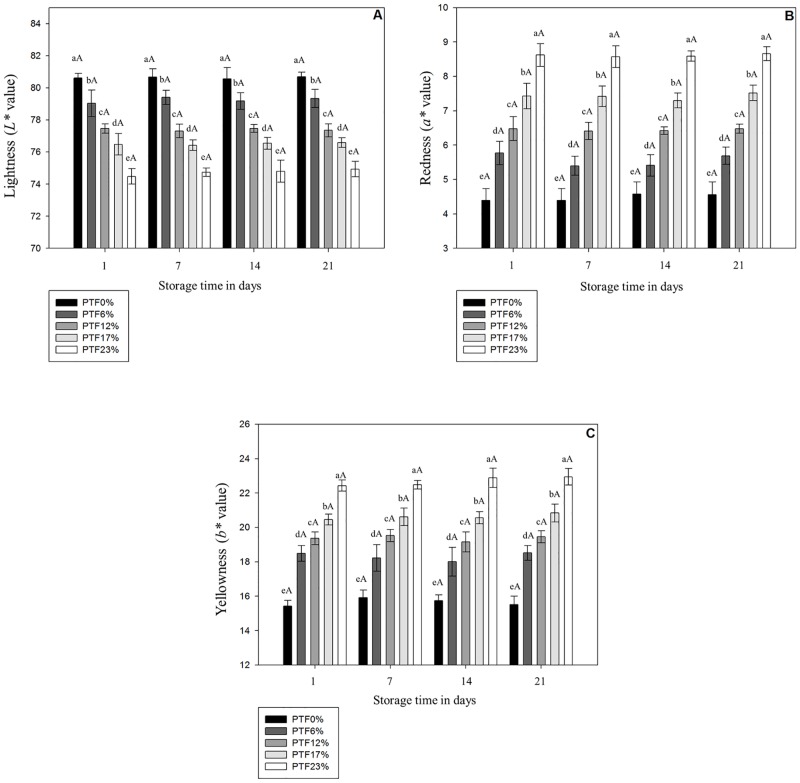
L*, a* and b* values in pasta fortified with different tilapia flour levels during 21 days at 25°C. PTF0%, PTF6%, PTF12%, PTF17%, and PTF23% mean pasta with 0%, 6%, 12%, 17%, and 23% (w/w) of tilapia flour, respectively. Results are expressed as means ± standard deviation (n = 3). Different lowercase letters indicate difference (*p* < 0.05) among formulations on the same day, and different uppercase letters indicate difference (*p* < 0.05) of means among storage days within the same formulation.

L*, a* and b* values are presented in Fig 1. The substitution of wheat flour by tilapia flour decreased (*p* < 0.05) the L* values (Fig 1A), and increased (*p* < 0.05) both a* (Fig 1B) and b* values (Fig 1C) of pasta formulations in a tilapia flour inclusion level-dependent manner. The heating during dry pasta manufacturing potentially promotes reactions between amine groups from protein and reducing ends of polysaccharides favoring Maillard reaction [48] potentially favoring the increase on the a* values [49,50] in formulations with increased tilapia flour content. Regarding b* values, pasta formulations were manufactured with white wheat flour and tilapia flour, which naturally presents a yellow color justifying a gradual increase in b* values with greater tilapia flour addition. On the other hand, lightness is influenced by water holding capacity and the amount of water on the foodstuff surface, therefore high water holding capacity decreases the free water content on the product’s surface resulting on lower L* values [51]. Furthermore, according to Svec et al. [52] color changes in pasta formulations depend on intrinsic characteristics of the original food matrix utilized for the wheat flour replacement. There is limited information regarding the instrumental color parameters of pasta enriched with fish therefore, our findings were compared to L*, a*, and b* values of pasta manufactured with other protein sources such as shrimp, corn and lupin protein isolates, and lentil and yellow pea flours. Similary to our results, a* and b* values were increased, while L* values were decreased in dry pasta added with shrimp meat [53], corn, and lupin protein isolates [52,54], and lentil and yellow pea flours [55].

No difference (*p* < 0.05) was observed in L*, a*, and b* values during the entire storage in all formulations. Pasta stability is directly affected by formulation and the drying procedure which together dictate the water dynamic in the final product [56,57]. Our findings are potentially associated with the low water activity hindering chemical reactions [58] and favoring a more stable matrix during storage [56]. Likewise, L* values did not change in storage period potentially due to very low water mobility in this type of product [59]. Regarding a* values, by reducing the water activity of foodstuff there is a mitigation of Maillard reaction [60] which is delayed and/or inhibited in a_w_ values bellow 0.64 [61]; in the present study all formulations exhibited a_w_ values lower than 0.6.

### Water Activity (a_w_) and pH

**Fig 2:**
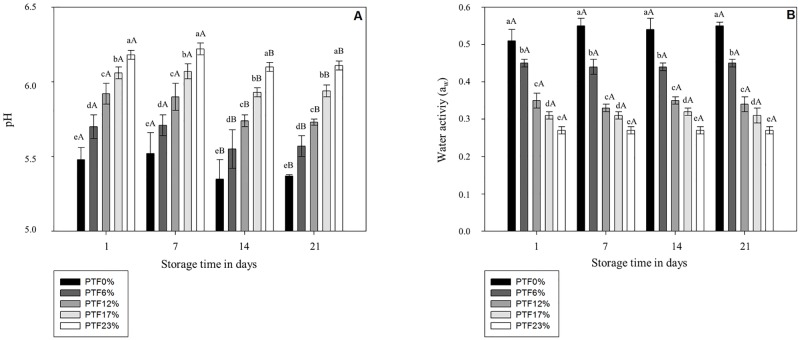
pH and water activity (aw) in pasta fortified with different tilapia flour levels during 21 days at 25°C. PTF0%, PTF6%, PTF12%, PTF17%, and PTF23% mean pasta with 0%, 6%, 12%, 17%, and 23% (w/w) of tilapia flour, respectively. Results are expressed as means ± standard deviation (n = 3). Different lowercase letters indicate difference (*p* < 0.05) among formulations on the same day, and different uppercase letters indicate difference (*p* < 0.05) of means among storage days within the same formulation.

The pH value (Fig 2A) increased (*p* < 0.05) whereas the a_w_ (Fig 2B) decreased (*p* < 0.05) as the level of tilapia flour increased in pasta formulations. There are no reports regarding pH and a_w_ in pasta formulations enriched with protein and lipid sources. Wheat flour exhibits pH values around 6.01 [62], while tilapia fillets exhibit a value closer to neutrality (6.35) [63] thus, the inclusion of tilapia flour promoted an increase on the pH value. Moreover, our study indicates that the gradual decrease of a_w_ in fish pasta formulations compared to control counterparts can be attributed to the capacity of protein to bind water molecules [64] and to the protein—polysaccharide—water complex favoring water entrapment [38,39]. Moreover, tilapia flour contains increased levels of lipid [11,35], which upon association with protein can interact with glutenin and gliadin from wheat culminating on a stronger matrix network [65].

Furthermore, the pH values decreased (*p* < 0.05) from day 7 of storage until the end of the storage period in all formulations. A decrease in the pH values during storage can be attributed to the formation of organic acids [66]. Similarly to our results, Pilli et al. [67] observed a trend of decrease on the pH values on dried pasta due to organic acids formation. In addition, no difference (*p* < 0.05) was found in a_w_ of pasta formulations due to stored during the 21 days at 25°C potentially due to the low water mobility in pasta products [59]. In partial agreement with our findings, Lodi et al. [68] observed homogeneous water distribution and minimal water loss and water migration in bread stored for 10 days.

### Lipid and Protein Oxidations

**Fig 3:**
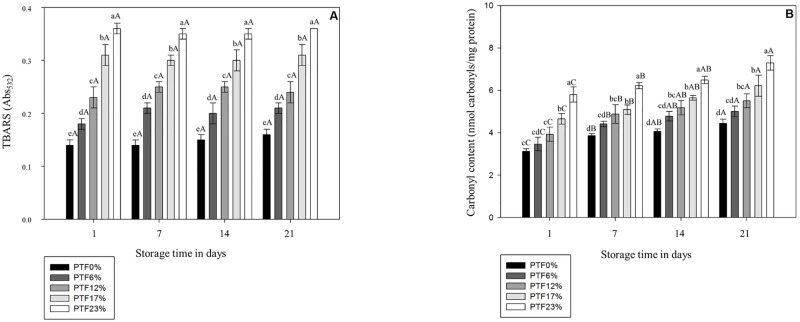
Thiobarbituric acid-reactive substances (TBARS) and carbonyl content in pasta fortified with different tilapia flour levels during 21 days at 25°C. PTF0%, PTF6%, PTF12%, PTF17%, and PTF23% mean pasta with 0%, 6%, 12%, 17%, and 23% (w/w) of tilapia flour, respectively. Results are expressed as means ± standard deviation (n = 3). Different lowercase letters indicate difference (*p* < 0.05) among formulations on the same day, and different uppercase letters indicate difference (*p* < 0.05) of means among storage days within the same formulation.

The replacement of wheat flour by tilapia flour promoted an increase (*p* < 0.05) on TBARS values in a tilapia flour inclusion level-dependent manner (Fig 3A). Fish contain high amount of polyunsaturated fatty acids (PUFA) which are more susceptible to lipid oxidation [69] supporting our findings for the TBARS values on day 1. The studies investigating lipid oxidation in pasta enriched with fish are limited, therefore our data were also compared to bakery products manufactured with soybean flour and flaxseed meal which are high in protein and PUFA, respectively [53,70]. These authors also observed an increase on lipid oxidation at the beginning of the storage period when bread flour was partially replaced by soybean flour and flaxseed meal. Furthermore, in agreement with the results of the present study, Kadam and Prabhasankar [53] reported greater lipid oxidation in pasta manufactured with shrimp meat than in control counterparts at the first day of storage.

In terms of protein oxidation, while PTF23% demonstrated the greatest (*p* < 0.05) carbonyl content (Fig 3B) amongst all formulations, no difference (*p* > 0.05) was observed between PTF17% and PTF12%, and between PTF12% and PTF6%. Whereas, PTF17% exhibited greater (*p* < 0.05) carbonyl content than PTF6%. Although carbonyl content was not affected (*p* > 0.05) by the inclusion of tilapia flour at 6%, at levels greater or equal to 12% it was observed an increased (*p* < 0.05) on protein oxidation in comparison to control after the 1^st^ day of storage. Nonetheless, there was a trend of greater carbonyl content following a inclusion level-dependent manner of tilapia flour on each storage point. Fish by-products exhibit a potential alternative to increase the protein levels in wheat-based products which are usually limited in such nutrient [8,15], however the increase on the protein level potentially favors oxidative reactions leading to the formation of carbonyl compounds [71]. Protein oxidation can occur via reaction with labile oxygen species derived from Maillard reaction, lipid peroxidation and/or autoxidation of sugars [72] favored by thermal processing [71] potentially explaining our results. There are no studies evaluating the protein oxidation in pasta fortified with protein and/or lipid, however several authors evaluated dried pasta nutritionally fortified with other protein and lipid sources as aforementioned, and observed an immediate increase in Maillard reaction and enhanced lipid oxidation [52–55,70), which potentially catalyze the protein oxidation [72]. The increased lipid and protein oxidation in pasta formulations supplemented with tilapia flour may be attributed to the higher amount of PUFAs and proteins which are more susceptible to lipid oxidation and carbonylation, respectively [69,71].

Throughout the storage, the carbonyl content increased (*p* < 0.05) in all formulations (Fig 3B). Although shelf-stability of powder products is predominantly dictated by a_w_, the physical and chemical reactions during storage depends on their chemical composition, manufacturing process, and external factors such as light exposure, oxygen permeability, and storage temperature [73]. Proteins and carbohydrates represent the main components in all pasta formulations on the present study, and both protein and amylose are susceptible to oxidative processes [71,74]. Nonetheless, the mechanisms involving changes in the starch component (amylose and amylopectin) of pasta products manufactured with functional ingredients are still unknown [56]. Moreover, carbonyl formation depends on the amino acid composition [75] which was variable in all formulations. To the best of our knowledge, there are no reports documenting the carbonyl content during storage at 25°C of regular pasta and pasta enriched with protein and lipid sources. Therefore, our results indicate that the increased carbonyl content during storage can be associated with the formation of lipid-protein and starch-protein arrangements due to different protein, lipid and carbohydrate ratios in each formulation.

Lipid oxidation was not influenced (*p* > 0.05) by the storage period (Fig 3A). Lipase and lipoxygenase are important enzymes involved with lipid oxidation in foods [76] that can be inactivated during heat processing wherein time, temperature and manufacture procedure are the main factors for their inactivation [77,78]. Moreover, lipid oxidation is enhanced in products exhibiting high and low a_w_ values with a lower reaction rate in the a_w_ range of 0.3–0.5, which includes most of the formulation investigated in the present study. Another potential explanation for the lipid oxidative stability during storage is related to the unavailability of lipid molecules due to their binding with proteins, carbohydrates or both compounds during extrusion preventing oxidation [43]. In agreement with our findings, Hall et al. [79] observed no variation on lipid oxidation values in dried flaxseed-added macaroni stored at room temperature. Verardo et al. [80] also reported lipid oxidation stability of spaghetti fortified with long chain n-3 PUFA stored for 3 months at room temperature and exposed to light. Therefore, our study indicates that the lipid stability of all formulations during storage was potentially associated with dry pasta manufacture where drying and extrusion processes lead to the decrease on oxidative processes involving lipids and to a decrease on water activity.

## Conclusions

The replacement of wheat flour by tilapia flour increased the nutritional quality of dried pasta including greater protein and lipid contents containing high quality essential fatty acid and amino acid profiles. Although some formulations of tilapia flour-enriched pasta exhibited an increase in the values of redness, yellowness, lipid oxidation and protein oxidation, the substitution of wheat flour by tilapia flour up to 6% can be considered a suitable strategy to improve the nutritional aspects of dried pasta without negatively affecting the storage quality of this product during at least 21 days at 25°C. Such strategy represents an alternative to the food industry to satisfy marketing trends and consumption patterns.
